# Impact of clinical preparation steps and use of sex-specific reference for accurate antibiotic monitoring in body fluids

**DOI:** 10.1038/s43856-025-00823-9

**Published:** 2025-04-16

**Authors:** Christian Domes, Lisa Graul, Timea Frosch, Juergen Popp, Stefan Hagel, Mathias W. Pletz, Torsten Frosch

**Affiliations:** 1 Biophotonics and Biomedical Engineering Group, Technical University Darmstadt, Merckstraße 25, 64283 Darmstadt, Germany; 2 Leibniz Institute of Photonic Technology, 07745 Jena, Germany; 3 Institute of Infectious Diseases and Infection Control, University Hospital, 07747 Jena, Germany

**Keywords:** Drug development, Biophysical methods

## Abstract

**Background:**

Effective antibiotic therapy in critically ill patients requires precise dosing tailored to individual conditions. However, physiological changes in these patients can complicate drug exposure prediction, leading to treatment failure or toxicity. Therapeutic drug monitoring (TDM) is crucial in optimizing antibiotic therapy, with Raman spectroscopy emerging as a promising method due to its speed and sensitivity.

**Methods:**

The utility of resonance Raman spectroscopy in analyzing clinical urine samples was investigated, specifically focusing on piperacillin concentrations. Samples subjected to various preparation techniques, including freezing, centrifugation, and filtration, were analyzed using deep UV resonance Raman spectroscopy. Data analysis involved preprocessing and chemometric modeling to assess concentration changes and the influence of sample matrix.

**Results:**

Sample preparation steps induce concentration changes in piperacillin, with freezing having the highest impact. Chemometric modeling reveals that freezing, filtration, and centrifugation, especially when combined, reduce drug concentration. Furthermore, the choice of urine reference for quantification impacts results, with sex-specific urine pools showing better accuracy compared to mixed pools.

**Conclusions:**

Resonance Raman spectroscopy effectively quantifies piperacillin concentrations in urine. Freezing, centrifugation, and filtration during sample preparation influence drug concentration. Using sex-specific urine pools as references yields more accurate quantification results. These findings underscore the importance of considering sample processing effects and reference selection in TDM studies, offering insights for optimizing antibiotic dosing in critically ill patients. Further validation on a larger scale is warranted to confirm these observations.

## Abstract (web summary)

Domes et al. use enhanced Raman spectroscopy to quantify drugs within body fluids. Freezing, centrifugation, and filtration result in additive loss of piperacillin from human urine, while using a reference urine pool from same-sex donors yield superior results to a mixed-sex urine pool.

## Plain language summary

It is important to dose people with an appropriate amount of a drug to ensure the drug can work effectively while minimizing side effects. By measuring the amount of antibiotic present in their urine, it is possible to determine whether the correct amount of an antibiotic drug has been given to a patient. The amount of antibiotics present in human urine was compared after various processing steps, such as filtering or freezing, which are often undertaken in hospital laboratories. The processing steps reduced the amounts of antibiotic present. It was also found that more accurate measurements of drugs in urine were made when body fluids from people of the same, rather than the opposite, sex were used as reference. In the future, this method could enable optimal, personalized treatment for various patient populations.

## Introduction

Piperacillin is a broad-spectrum β-lactam antibiotic with high efficacy against gram-positive and gram-negative aerobic and anaerobic bacteria^1,2^. It is used to treat a variety of infections, including severe pneumonia^3^ and urinary tract infections^4^.

For successful treatment of critically ill patients, antibiotic therapy should be individually tailored to the prevailing physical conditions, including consideration of organ function^5,6^. However, the pathology of critically ill patients may cause physiological changes that grossly alter the pharmacokinetics of antimicrobial substances^1,7^ and complicate the prediction of drug exposure^8,9^. These physiological changes can result in underdosing with a risk of treatment failure and overdosing with a risk of accumulation and toxic adverse effects. Above all, in vitro data show that subinhibitory antibiotic concentrations can foster multidrug resistance^10–12^. Therapeutic drug monitoring (TDM)-guided antibiotic therapy may be a proper strategy to overcome this pharmacokinetic variability and optimize target attainment^13^. The most commonly used techniques for TDM are based on chromatography, e.g., liquid chromatography tandem mass spectrometry (LC-MS/MS)^14,15^ and (ultra)high-performance liquid chromatography (UHPLC^(^™^)^)^16–18^, which are highly sensitive and specific; however, these techniques are time consuming and labor-intensive and waste organic solvents. Raman spectroscopy^19,20^ is a non-invasive, label-free, fast, and sensitive technique^21–24^ for chemical selective multicomponent analysis^25–27^ and, therefore, a promising method for TDM^28,29^. Recently, several advanced techniques, such as fiber enhanced Raman spectroscopy (FERS^30–36^), surface enhanced Raman spectroscopy (SERS^37–39^), and resonance Raman spectroscopy (RRS^40,41^), have been developed to enhance Raman signals for sensitive monitoring of drug concentrations. For the quantification of dissolved antibiotics, their matrix, i.e., the body fluid, has a major impact due to its comparatively high background signals and must therefore be considered. This effect can be compensated either by upstream analyte separation (mainly in SERS^42–44^) or by chemometric data preprocessing^45,46^.

In this study, the precise analysis and consideration of antibiotics concentration changes resulting from the clinical sample preparation steps, such as freezing, centrifugation, and filtration, are thoroughly investigated. Deep UV resonance Raman spectroscopy and chemometric modeling are used to assess concentration changes and the influence of the sample matrix. It can be shown that piperacillin concentrations are reduced by sample preparation steps, with freezing having the highest impact. Using sex-specific urine pools as a reference matrix for drug quantification is shown to provide better accuracy compared to mixed pools.

## Methods

### Sample preparation

Piperacillin (piperacillin sodium salt, 90%, Santa Cruz Biotechnology Inc., Dallas, Texas USA) was used without further purification. For urine samples, midstream urine was collected from healthy non-smoking volunteers between 18 to 30 years in the morning. The ethical compliance approval was provided by Ethics Committee of the University Jena (#5063 - 02/17, with amendments). Before providing urine samples, all individual volunteers received a comprehensive oral description of the experiments and provided their consent to the procedure. The urine samples were anonymized, only the donors’ sex was specified. The samples were centrifuged [425 × *g* for 10 min (XC-2009, Premiere Centrifuges, Morgan Hill, CA USA)] and sterile filtrated (sterile syringe filter with a pore diameter 0.22 µM, VWR International, Dresden, Germany).

Female, male, and mixed urine pools were prepared by equally mixing all female (*n* = 8), male (*n* = 9), and all urine samples, respectively. Urine samples were measured shortly after collection, and after, they were stored in the freezer at −80 °C.

To investigate the effect of sample preparation, urine samples used for calibration were centrifuged and/or filtered or frozen and spiked with piperacillin at various concentrations. The samples used for evaluating the different preparation effects were first spiked with the same piperacillin concentrations, treated accordingly, and later evaluated similar to the respective calibration groups.

### Spectroscopy

Deep UV resonance Raman spectroscopy (DUV-RR) spectra were acquired with a Raman spectrometer (LabRAM Jobin Yvon) equipped with a UV laser (Innova, Coherent). The excitation wavelength λ_exc. _= 244 nm was focused on the sample (laser power: approximately 1.3 mW) and collected with the same objective lens. Using a 2400 l/mm grating and an exposure time of 30 s (two accumulations), three (20) spectra were measured from each sample for quantification (classification). To avoid any photodegradation during the measurements, the samples were measured in a rotational cuvette.

### Data analysis

All preprocessing and analysis of the raw Raman data was performed in the statistical programming GnuR 3.5.0^47^. The packages ‘minpack.lm’^48^, ‘signal’^49^, ‘EMSC’^50^, ‘Peaks’^51^, ‘pls’^52^, ‘MASS’^53^, ‘e1071’^54^, and ‘ROCR’^55^ were utilized, and their functions were implemented *via* an in-house written procedure. In all cases, a wavenumber calibration was performed using the Raman signals from acetonitrile, Teflon, and toluene, which were measured before the experiment and calibrated to their respective reference spectra. Then, the Raman data were truncated to the wavenumber region of interest (1800 cm^−1^–800 cm^−1^), and Savitzky-Golay smoothing (*p* = 2, *n* = 5) was applied. Subsequently, the resulting spectra were scatter corrected for the median spectra of each sample using the extended multiplicative signal correction (EMSC) procedure (degree = 6) and baseline corrected using the SNIP algorithm (iterations = 20, order = 2)^56^.

**Fig. 1:**
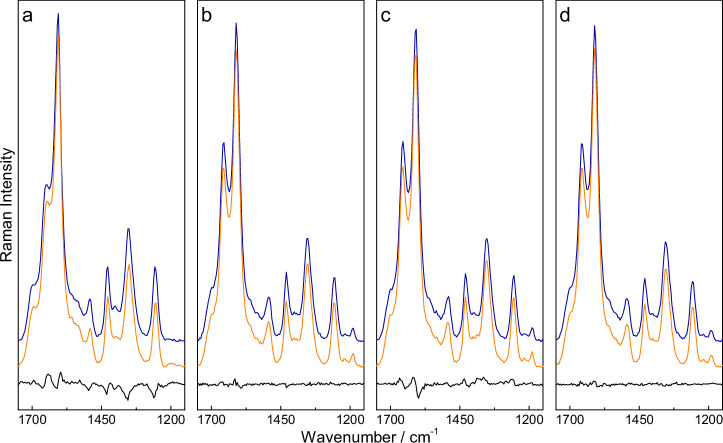
Variations in piperacillin Raman spectra in human urine due to sample treatment. Representative vector normalized median Raman spectra of a 1000 µM solution of piperacillin in human urine before (orange) and after treatment (blue) and their difference (black). The effects of freezing (**a**), filtration (**b**), centrifugation (**c**), and a combination of filtration and centrifugation (**d**) were investigated, showing only small changes in the overall spectra and signal intensities.

**Fig. 2:**
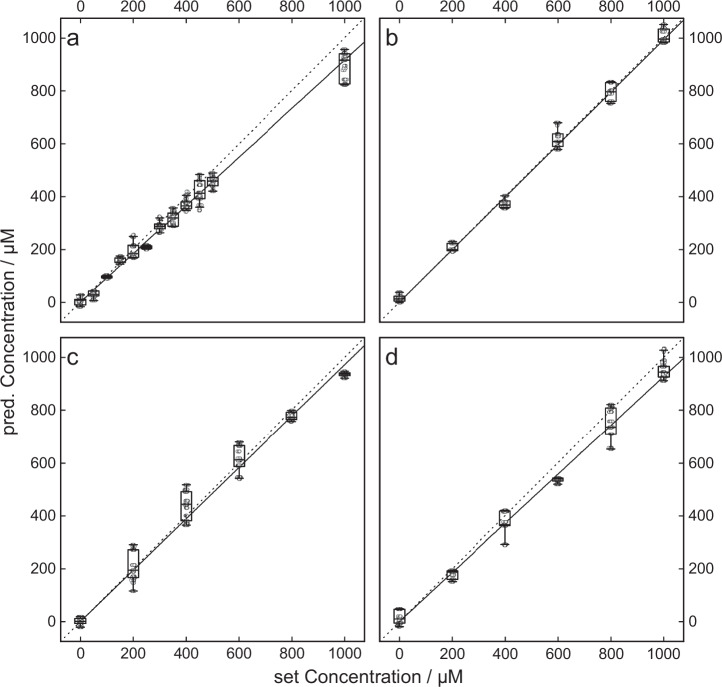
Influence of sample preparation techniques on piperacillin concentrations. Comparison of the piperacillin concentrations of the untreated and treated samples for the individual sample preparation steps [i.e., freezing (**a**), filtration (**b**), centrifugation (**c**), and filtration combined with centrifugation (**d**)]. The concentrations of the treated samples were determined through a PLS regression model based on the untreated samples. The correlation between these concentration values indicates the influence of individual sample preparation steps. The factor (see Table 1) was calculated by minimizing the RMSEP of the concentrations of the respective treated and untreated samples [see Eq. (1)], resulting in an almost perfect linear relationship (identity function, dashed line). Filtration and centrifugation have a small effect on the concentration change of piperacillin, while freezing shows the largest effect. The boxplot shows the predicted concentrations resulting from a 50-fold cross-validation of *n* = 3 replicate measurements. In addition, all obtained measurements are shown, jittered for better visualization.

First, the influence of routine sample treatment steps, i.e., freezing, filtration, centrifugation, and the combination of centrifugation and filtration, on the concentration of piperacillin in urine was investigated. For this purpose, urine samples were first treated and subsequently spiked with known antibiotic concentrations (reference), while samples of known concentration were spiked prior to treatment (sample). Based on the respective references, a PLS model was created, whereby the previously treated Raman spectra were truncated to a smaller wavenumber range (1750 cm^−1^–1150 cm^−1^) and vector normalized (see Fig. 1). The reference data was then divided into training and test data (80% and 20%) and the optimal number of three to five components was calculated by minimizing the root-mean-square error of prediction (RMSEP) using 50-fold cross-validation. This regression model was then used to determine the unknown concentrations of the samples (see Fig. 2).

**Fig. 3:**
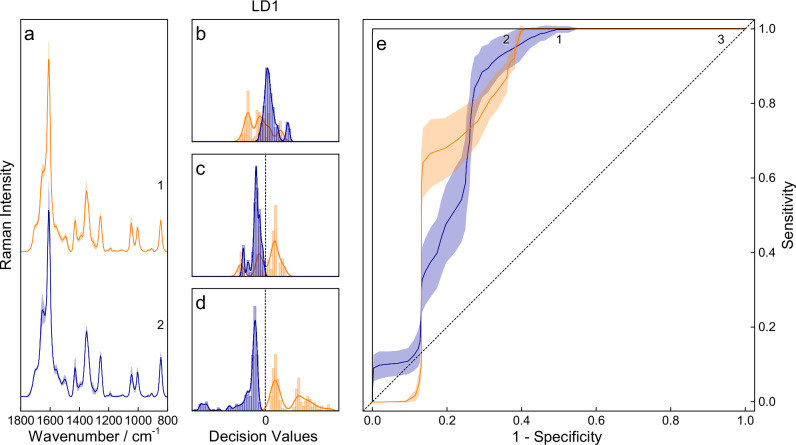
Raman spectroscopic analysis of urine samples for sex-classification. Median resonance Raman spectra with the respective standard deviation of female (orange, **a**1, *n* = 8) and male (blue, **a**2, *n* = 9) urine from healthy volunteers using *λ*_exc._ = 244 nm as the excitation wavelength. Different classification algorithms, i.e., LDA (**b**) and SVM with linear (**c**) and radial (**d**) kernels, are applied, and their respective histograms with smoothed kernel distribution are shown. LDA and linear SVM show similar results with accuracies of approximately 75%. Since this classification problem seems to be nonlinear, the radial kernel SVM can correctly classify both groups. Additionally, the threshold-averaged (solid) ROC curves of all algorithms (**e**) show the same behavior for the LDA (blue, **e**1) and SVM [linear (orange, **e**2) and radial (black, **e**3)] classifiers. The solid lines represent the mean values, while the shaded areas correspond to the standard deviation, calculated from *n* = 20-fold cross-validation.

For classification of the urine samples of different sexes, the baseline corrected truncated data were vector normalized, and principal component analysis (PCA) was utilized for dimension reduction, with five components covering 95% of the cumulative expressed variance. Subsequently, the reduced data set was divided into a training set and a test set (80% and 20%), and classification was performed using linear discriminant analysis (LDA) and a supporting vector machine (SVM) with linear (cost = 1000) and radial (cost = 10,000, γ = 1) kernels. The results were 20-fold cross-validated, and a receiver operation characteristic (ROC) analysis was performed (see Fig. 3).

**Fig. 4:**
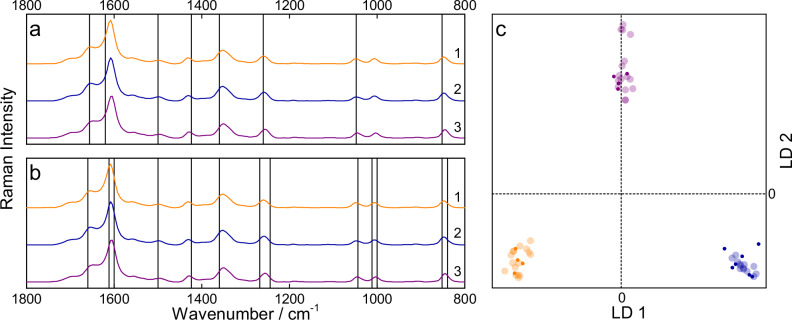
Raman spectroscopic analysis of sex-specific differences in urine pools. Median resonance Raman spectra of female (orange, 1), male (blue, 2), and mixed (purple, 3) pools with the weighting vectors for LD1 (**a**) and LD2 (**b**) depicted as vertical lines, showing the extrema of the discriminant coefficient scaled PCA loadings. Specifically, the lines at approximately 850, 1050, and 1525 cm^−1^ can be assigned to creatinine, while the lines at 1015, 1265, and 1611 cm^−1^ and at 1355, 1435, and 1660 cm^−1^ originate from urea and uric acid, respectively. With this linear classifier, the data can be perfectly discriminated (**c**). The test and training scores of LD1 and LD2 are depicted as small and large transparent dots, respectively.

To examine the impact of urine donor’s sex as a reference on the quantification of antibiotic concentration in urine samples, three different pools were prepared. To distinguish between these mixtures, an LDA procedure was applied in which the aforementioned pre-processed data set was vector normalized, dimension reduced via PCA (15 PCs), and divided into training data and test data (80% and 20%). The weighting vector (scaled loading vector) was calculated to determine which structural part of the molecule was responsible for the discrimination. The main differences resulted from the specific distribution of creatinine, urea, and uric acid in the samples (see Fig. 4)^57^.

**Fig. 5:**
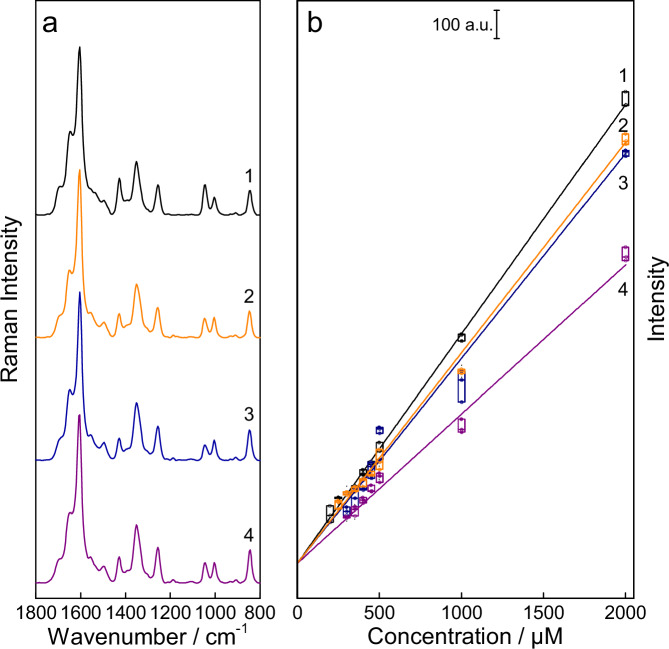
Impact of reference on piperacillin quantification in female urine using Raman spectroscopy. Median resonance Raman spectra of the female’s own urine (black, **a**1) as well as the female (orange, **a**2), male (blue, **a**3), and mixed (purple, **a**4) urine pools. These spectra were further used as a matrix for the quantification of piperacillin in female urine. The calibration curves using the female’s own urine (black, **b**1), female (orange, **b**2), male (blue, **b**3), and the mixed urine pool (purple, **b**4) as references show slight changes in their slope, while the female’s own urine leads to the lowest LoD, followed by the female pool, mixed pool, and male pool regarding their sensitivity. Despite the high slope, the high standard error of the axis intercept results in a worse LoD for the male pool [see Eq. (2)]. The boxplot represents the predicted intensity values of *n* = 3 replicate measurements.

For quantification of the reference effect, the spectra were maxima normalized at ~1605 cm^−1^ (see Fig. 5a) depicted as the C=O-stretching vibration of urea^58^. The difference spectra of the urine samples spiked with piperacillin and the pure urine samples were calculated using the respective median urine spectra as a reference, and a Gaussian peak profile was fitted to the HC–CH-rocking and NH-out-of-plane bending vibration of piperacillin at ~1485 cm^−1^. The resulting peak intensities were correlated with the corresponding concentrations, showing a linear relationship (see Fig. 5b). A detailed characterization of the vibrations of the β-lactam piperacillin could be found in a previous study^40^.

### Reporting summary

Further information on research design is available in the Nature Portfolio Reporting Summary linked to this article.

## Results

Regarding the investigation of clinical samples, analyte quantification is of major research interest. In this context, Raman spectroscopy and its enhancement techniques have been more frequently used for TDM in body fluids^28,29,59–61^. This study aims to determine the effects of sample processing on the analyte concentration and the choice of proper reference for quantification approaches.

### Induced concentration changes due to sample preparation

In routine clinical practice, patient samples are frozen for storage and/or centrifuged and filtered to remove cells and debris that would interfere with further investigation after collection. These sample treatments are accompanied by a change in drug concentration; however, to the best of the author’s knowledge, the freezing effect has only been investigated^62,63^.

The concentration change can be verified using representative spectra of a 1000 µM solution of piperacillin in human urine, where each preparation step causes small changes in the shape of the spectra (see Fig. 1).

To quantify the preprocessing factor, a concentration series of untreated and treated samples (piperacillin in female urine) were prepared; the former was used to predict the latter via PLS regression.

The predicted concentration values were compared with the theoretically adjusted values, and the root mean square error of prediction (RMSEP) between these values was calculated. Then, a factor was applied to the respective predicted concentrations, which minimizes this measure using Eq. (1):

1 $${{{{\rm{argmin}}}}}_{f}\sqrt{\frac{1}{n}\cdot {\sum }_{i=1}^{n}{\left({c}_{{{{\rm{set}}}},i}-\frac{{c}_{{{{\rm{pred}}}},i}}{1-f}\right)}^{2}}$$

where ${c}_{{{{\rm{set}}}},i}$ and ${c}_{{{{\rm{pred}}}},i}$ are the set and predicted concentrations, respectively, and $f$ is the scaling factor (see Fig. 2). For this method, the effect of freezing had the highest impact (8.42%), while filtration (2.36%) and centrifugation (2.80%) had lower effects on the concentration of analytes but were additive when performed sequentially [5.05% (see Table 1)]. These factors needed to be calculated for each drug and matrix but could then be used to compensate for the investigated effects of sample treatment.

### Effect of urine matrix on drug quantification

A further step towards the analysis of clinical urine samples is the investigation of the influence of the matrix of the analyte. Since many studies have analyzed urine Raman data^64–66^, this study aims to investigate the impact of the different references on the quantification of the solute, i.e., piperacillin.

The impact of the donor’s sex was investigated by examining the urine samples from 17 healthy volunteers using LDA and SVM with linear and radial kernels (*vide supra*, see Fig. 3a). The data were divided into training data and test data; the test data were classified using the trained model, and 20-fold cross-validation was applied. Then, an ROC was used as a measure of the goodness of classification (see Fig. 3e), and the area under the curve was calculated for each individual classification. The values for LDA, linear SVM, and radial SVM were 0.80 ± 0.05, 0.81 ± 0.05, and 1.00 ± 0.00, respectively. In this context, a value of 1.0 is equivalent to a perfect classification, and 0.5 is a random choice^67^. Comparing the numbers and histograms of the individual classification models (see Fig. 3b–d) clearly indicates a nonlinear classification problem, which explains the comparatively poor performance of LDA and SVM with a linear kernel. However, a nonlinear classification model could effectively distinguish the urine samples of different sex-specific donors.

If the patient’s untreated own urine is not available and therefore cannot be used as a reference for quantification, the concept is to prepare a mixture of different urine samples, a so-called pool, which can be used as a substitute. Since the previous experiment showed that the sex of the urine donors had an impact on the discrimination of the respective samples, a classification algorithm was also applied to the spectra of the three different pools.

This time, all three pools could be perfectly discriminated using the LDA model, with the different composition of the pooled samples; the concentrations of urea, creatinine, and uric acid had the major effect (see Fig. 4).

The impact of sex on the quantification of piperacillin was investigated by preparing a female, a male, and a mixed pool (*vide supra*) as individual references (see Fig. 5a). Then, the marker band at 1485 cm^−1^ was fitted with a Gaussian peak profile, and its intensities were correlated with the respective concentration. The limit of detection (LoD) and quantification (LoQ) were calculated according to the International Council for Harmonization (ICH) guidelines^68^ using Eq. (2):

2 $${LoD}\,\left({LoQ}\right)=3.3\left(10\right)\cdot \frac{{\sigma }_{{{{\rm{n}}}}}}{m}$$

where ${\sigma }_{{{{\rm{n}}}}}$ and $m$ are the standard error of the intercept and the slope of the quantification curve, respectively (see Fig. 5b).

For the female samples, the results were almost the same when using the female’s own urine as a reference compared to the female pool (deviation of LoD of 11%). The mixed pool showed a worse deviation (25%) but was still better than using the male pool (57%, see Table 2). Therefore, it is recommended to use a sex-specific pool if the original pool is not available to ensure comparable quantification results.

## Discussion

Precise analyte quantification in clinical samples is crucial for various research endeavors. Raman spectroscopy and its enhancement techniques have gained prominence in TDM within body fluids, reflecting a growing interest in its application. This study seeks to elucidate the impact of sample processing on analyte concentration and the selection of suitable references for quantification methods.

The observed drug losses resulting from sample preparation procedures, i.e., freezing, centrifugation, and filtration, underscore the need for meticulous consideration in clinical practice. While freezing has been previously examined, this study delves deeper into the combined effects of multiple processing steps. Representative Raman spectra of piperacillin in human urine highlight subtle alterations induced by each preparation method. Through predictive modeling using PLS regression, a factor is determined to mitigate these effects, with freezing demonstrating the most impact followed by filtration and centrifugation. These findings emphasize the necessity of accounting for sample treatment effects to ensure accurate quantification.

Moreover, the influence of urine matrix on drug quantification is explored, particularly focusing on the selection of appropriate references. Sex-specific variations in urine composition are elucidated through classification algorithms, revealing the nonlinear nature of the classification problem. When untreated own urine is unavailable, pooling strategies are commonly employed, albeit with consideration of donor sex. The investigation highlights the superiority of sex-specific pools over mixed pools ensuring robust quantification outcomes. This comprehensive analysis underscores the importance of meticulous sample processing and reference selection in TDM studies, offering valuable insights for optimizing analytical protocols in clinical settings.

In this study, the utility of resonance Raman spectroscopy for the analysis of clinical urine samples was investigated. Using the DUV excitation wavelength *λ*_exc._ = 244 nm, the detection of clinically relevant concentrations^69^ was possible with small sample volumes (400 µL) and short measurement times (60 s). Furthermore, the effects of different sample preparation steps in routine clinical practice, such as freezing, centrifugation, and/or filtration, on the concentration of piperacillin were determined. Freezing had the highest effect on the piperacillin concentration, while the effects from the other techniques were lower; however, when performed sequentially with both centrifugation and filtration, the effects were additive, which should be considered in future quantification analysis.

**Table 1:** Correction factors for sample preparation

Preparation method	Factor/%	RMSEP/µM	RMSEP*/µM
froz	8.42 ± 0.04	57.8	10.4
filt	2.36 ± 0.04	14.7	14.1
cent	2.80 ± 0.10	33.5	29.8
filt + cent	5.05 ± 0.06	45.4	12.1

Correction factor for the effect of freezing (froz), centrifugation (cent), filtration (filt), and their combination (filt + cent) using PLS regression model. Since the factor evaluation is based on the RMSEP, this value is also listed before and after (*) minimization. Freezing has the highest effect on concentration changes, centrifugation and filtration are lower, but when applied sequentially, they are additive. The error of the respective factor results from the 10-fold cross-validation of the predicted data. In each case, 80% of the data was used and the factor was calculated according to Eq. (1). The specified value corresponds to the median and the respective median absolute deviation.

**Table 2:** Comparison of piperacillin’s detection limit using different reference samples

Reference	LoD/µM (mg/L)	LoQ/µM (mg/L)
own urine f	27.3 (14.7)	82.8 (44.7)
pool f	30.9 (16.7)	93.5 (50.4)
pool m	64.0 (34.5)	194 (105)
pool mix	36.6 (19.7)	111 (59.9)

Comparison of the limit of detection and quantification (LoD and LoQ) of piperacillin using different references. In all cases, the values were within the range of clinically relevant concentrations (25,238 ± 15,200) µM [(13,062 ± 7867) mg/L]. Since the dilution series was prepared in female urine, the female’s own urine and female pool provided the lowest LoD. Using a male and mixed pool, the LoDs increase by 25% and 57%, respectively. It is therefore recommended to use a sex-specific pool if the original pool is not available.

Subsequently, the choice of urine from different donors as a reference for quantification was investigated. For this purpose, their pool was classified in advance according to sex, and a difference between male and female donors and their respective (mixed) pools was found. This was also confirmed during quantification; the sex’s own urine sample and the corresponding sex-specific urine showed comparable results, whereas the use of sex-nonspecific (opposite) urine and the pooled mixture as a reference were not recommended due to their higher uncertainties. However, considering the limited number of donors in each group, this aspect should be verified and validated on a larger scale.

In summary, the results achieved can be used to better understand the quantification challenges and their compensation when dealing with clinical samples and serve as a foundation for future clinical studies for the inclusion and comparison to other antibiotics.

## Supplementary information

Supplemental Data 1

Supplemental Data 2

Supplemental Data 3

Supplemental Data 4

Supplemental Data 5

Reporting summary

## Data Availability

All figures were visualized using Origin 2015 software. The numerical data (source data) underlying Figs. 1–5 can be found in Supplementary Data 1–5.

## Code availability

All preprocessing and analysis of the raw Raman data was performed in the statistical programming GnuR 3.5.0^47^. The packages ‘minpack.lm’^48^, ‘signal’^49^, ‘EMSC’^50^, ‘Peaks’^51^, ‘pls’^52^, ‘MASS’^53^, ‘e1071’^54^, and ‘ROCR’^55^ were utilized.
